# Resistance of *Neisseria gonorrhoeae* isolates to beta-lactam antibiotics (benzylpenicillin and ceftriaxone) in Russia, 2015–2017

**DOI:** 10.1371/journal.pone.0220339

**Published:** 2019-07-25

**Authors:** Boris Shaskolskiy, Ekaterina Dementieva, Ilya Kandinov, Marina Filippova, Natalia Petrova, Xenia Plakhova, Alexander Chestkov, Alexey Kubanov, Dmitry Deryabin, Dmitry Gryadunov

**Affiliations:** 1 Engelhardt Institute of Molecular Biology, Russian Academy of Sciences, Moscow, Russia; 2 State Research Center of Dermatovenerology and Cosmetology, Russian Ministry of Health, Moscow, Russia; Emory University School of Medicine, UNITED STATES

## Abstract

The goal of this work was to study the phenotypic susceptibility and resistance determinants of *N*. *gonorrhoeae* isolates to beta-lactam antimicrobials (benzylpenicillin and ceftriaxone). A total of 522 clinical isolates collected in Russia in 2015–2017 were analysed for susceptibility using the agar dilution method. DNA loci involved in antimicrobial resistance were identified using DNA microarray analysis and sequencing. Resistance to benzylpenicillin remained high, with 7.7% of isolates resistant (MIC_pen_ > 1 mg/L) and 47.5% of isolates showing intermediate susceptibility (MIC_pen_ = 0.12–1 mg/L). The most frequent resistance determinant (72.4% isolates) was the Asp345 insertion in *penA*, both as a single mutation and in combination with other mutations, particularly with the substitution Leu421Pro in *ponA* (39.0%). Mutations affecting the influx and efflux of drugs were also found, including amino acid substitutions in PorB (26.8% isolates) and delA in the promoter region of *mtrR* (22.8%). The accumulation of mutations in chromosomal genes (*penA*, *pon*, *porA*, and *mtrR*) led to a stepwise increase in MIC_pen_ to values characteristic of intermediate resistance. The presence of *bla*_TEM_ plasmids was found in 25 isolates (4.8%), resulting in a strong increase in resistance to penicillin (MIC_pen_ > 16 mg/L) compared with the chromosomal mutations; 23 plasmids were of the African type with TEM-1 beta-lactamase, and two plasmids were of the Toronto/Rio type with TEM-135 beta-lactamase. Only three isolates were found with reduced susceptibility to ceftriaxone, with MIC_cef_ = 0.12–0.25 mg/L. Sequencing of *penA* did not reveal mutations associated with resistance to third-generation cephalosporins, and the gene structure was non-mosaic. The majority of isolates (21 of 25) carrying the *bla*_TEM_ plasmid also contained the conjugative plasmid with *tetM* (resistance to tetracyclines), consistent with previously reported data that the presence of the conjugative plasmid facilitates the transfer of other plasmids associated with antimicrobial resistance.

## Introduction

Gonorrhoea is a sexually transmitted infection caused by the gram-negative bacterium *Neisseria gonorrhoeae*. A distinctive feature of *N*. *gonorrhoeae* is its ability to rapidly accumulate different mutations to acquire resistance against the antibiotics used for its treatment [1,2]. The WHO has declared drug resistance in *N*. *gonorrhoeae* to be an emerging threat that has the potential to move gonorrhoea to the category of incurable infections [3].

Benzylpenicillin, a beta-lactam antibiotic, along with penicillins of subsequent generations, was used in the Russian Federation until the beginning of the 21st century. However, due to the development of a high resistance level in the population, it is no longer used for gonorrhoea treatment. At present, the antibiotics recommended for the treatment of gonorrhoea in Russia are ceftriaxone, a 3rd-generation cephalosporin, and spectinomycin. Unlike in European countries, azithromycin has never been recommended for gonorrhoea treatment in Russia, and cefixime has not yet been introduced into medical practice.

*N*. *gonorrhoeae* isolates that demonstrate resistance to 3rd-generation cephalosporins have recently appeared all over the world, including the H041 and F89 isolates, with MIC_cef_ ≥ 1 mg/L [4–8]. The susceptibility level of *N*. *gonorrhoeae* to beta-lactam antibiotics is under constant surveillance in the Russian Federation [9–11].

The molecular determinants associated with the resistance of *N*. *gonorrhoeae* to penicillins involve both chromosomal mutations and the presence of the *bla*_TEM_ plasmid encoding beta-lactamases (penicillinases) [1,4,12]. The chromosomal determinants include mutations in *penA* that result in a decrease in the affinity of the penicillin-binding protein (PBP2), such as the insertion of an Asp codon between positions 345 and 346 (insAsp345); mutations in the C-terminal region of PBP2 have also been described [13,14]. PBP2 types are designated by Roman numerals from I to XXXVIII based on substitution profiles at 82 amino acid positions [15–17]. The recently developed NG-STAR program (https://ngstar.canada.ca) summarizes the currently known PBP2 types. NG-STAR classification uses the entire *penA* sequence and combines the historical nomenclature for *penA* types with novel nucleotide sequence designations. It currently includes 49 *penA* types, 21 historical and 28 novel amino acid profiles, and 80 *penA* alleles [18].

Mutations in *ponA*, which encodes penicillin-binding protein 1 (PBP1), *e*.*g*., the Leu421Por substitution, lead to a decrease in the rate of penicillin acylation [19]. Mutations causing an increase in the expression of the MtrCDE efflux pump also result in increased penicillin resistance; the main mutations are insertions of T and TT and deletion of A in the promoter region of *mtrR*. The Gly45Asp substitution in the coding region of *mtrR* is much less frequent [20,21]. Mutations in *porB*, which encodes the porin protein PorB1b, at residues Gly120 and Ala121 [22] in the presence of mutations in *mtrR* [19] result in a change in the permeability of the cell membrane and a decrease in the influx of antimicrobials into bacterial cells. The accumulation of mutations leading to an increase in the MIC_pen_ has also been described; *N*. *gonorrhoeae* isolates with increased MIC_pen_ values of up to 1.0 mg/L were obtained in the laboratory by the stepwise addition of mutations in *penA*, *ponA*, *mtr*, and *porB* [19].

Plasmid-mediated TEM beta-lactamases catalyse the hydrolysis of the cyclic amide bond of penicillin, resulting in degradation of the antibiotic. The family of *N*. *gonorrhoeae bla*_TEM_ plasmids includes the following types: Asian (7426 bp), African (5599 bp), Toronto/Rio (5154 bp), Nimes (6798 bp), New Zealand (9309 bp), Johannesburg (4865 bp) and Australian (3269 bp) [23–26]. The Asian plasmid is considered to be a general ancestor from which plasmids of other types evolved by means of deletions and insertions [23, 25]. Five variants of plasmid beta-lactamases are known: TEM-1 (plasmids of all types), TEM-135 has the Met182Thr change in the amino acid sequence of the protein (usually typical of Toronto/Rio plasmids), TEM-220 contains the Met182Thr and Ala185Thr substitutions (Toronto/Rio plasmids), and enzymes with Glu110Lys and Gly228Ser substitutions occur among African plasmids [24, 26]. Although the described *N*. *gonorrhoeae* beta-lactamases cannot destroy third-generation cephalosporins, the emergence of extended-spectrum cephalosporin resistance in *N*. *gonorrhoeae* isolates is worrisome. TEM-135 beta-lactamase differs from TEM-1 by a single nucleotide (T→C in position 539, leading to the amino acid substitution Met182Thr). One additional specific SNP may lead to the Gly238Ser substitution, thus changing TEM-135 into the TEM-20 beta-lactamase, which is capable of destroying extended-spectrum cephalosporins [27,28].

The genetic determinant that is most often associated with *N*. *gonorrhoeae* resistance to cephalosporins is a mosaic structure of *penA*, which results from interspecies genetic recombination among *N*. *gonorrhoeae*, *N*. *cinerea* and *N*. *perflava* [16, 17]. Mosaic alleles can contain more than 70 amino acid changes compared with the wild-type protein that influence acylation by PBP2 [1]. Cephalosporin-resistant isolates with mosaic *penA* alleles often do not harbour the Asp345 insertion, which provides resistance to penicillins [16,29]. The Gly545Ser, Ile312Met, Val316Thr [30], Gly542Ser, Pro551Ser and Pro551Leu substitutions [31] in mosaic PBP2 genes have been suggested as mutations affecting cephalosporin resistance, but their role is not fully confirmed. Resistance to cephalosporins is also associated with non-mosaic alleles carrying substitutions of the Ala501 residue and mutations in *mtrR* and *porB* causing increased efflux and decreased influx of antimicrobials [5,20,22]. For example, two *N*. *gonorrhoeae* strains with high-level resistance to 3rd-generation cephalosporins that were isolated in Europe have a mosaic *penA*, type XXXIV, with an additional Ala501Pro substitution [32,33].

The goal of this work was to study the susceptibility of the current population (2015–2017) of *N*. *gonorrhoeae* isolates from the Russian Federation to beta-lactam antibiotics and to identify genetic determinants of resistance to these drugs, including investigation of the types of *bla*_TEM_ plasmid genes and beta-lactamase variants.

## Materials and methods

### *N*. *gonorrhoeae* clinical isolates

According to the Ethics Committees of the State Research Center of Dermatovenerology and Cosmetology, this research does not require ethical approval. All specimens used in this study were anonymous samples that omitted personal information about the patients, particularly their name or address.

*N*. *gonorrhoeae* clinical isolates were collected by the State Research Center of Dermatovenerology and Cosmetology, Russian Ministry of Health, Moscow, within the framework of the Russian Gonococcal Antimicrobial Surveillance Programme (RU-GASP) [9,10]. The collection included 522 isolates obtained in 2015–2017 from 16 regions of the Russian Federation, with centres in Arkhangelsk, Astrakhan, Bryansk, Cheboksary, Chelyabinsk, Irkutsk, Kaluga, Kazan, Moscow, Novosibirsk, Omsk, Penza, Pskov, Ryazan, Stavropol, and Tomsk (S1 Table).

The samples were obtained from clinical specimens (urethral specimens from men and cervical/urethral specimens from women) of patients with diagnosed primary symptomatic uncomplicated gonorrhoea who attended specialized dermatovenereological clinics. The patients had not used antibiotics for the treatment of gonorrhoea or other diseases during the last 12 months.

Primary *N*. *gonorrhoeae* identification was performed in regional clinics using Gram staining and the rapid oxidase reaction. Gram-negative and oxidase-positive culture samples were frozen in cryomedium-Trypticase soy broth containing 20% glycerol (Becton, Dickinson BBL, Sparks, MD, USA) and transported on dry ice to the State Research Center of Dermatovenerology and Cosmetology, Moscow. The cultures were then plated on GC-agar enriched with 1% IsoVitaleX and 1% VCAT selective supplement (Becton Dickinson, USA) and verified by tests for biochemical activities with NH ID cards on a VITEK 2 Compact Analyser (bioMérieux, France). For the cultures identified as *N*. *gonorrhoeae* with a probability of less than 95%, mass spectrometric studies were carried out using a MALDI Microflex (Bruker Daltonics GmbH, Germany).

The cultures were preserved in Trypticase soy broth with 20% glycerol at –70°C. Isolation of DNA from *N*. *gonorrhoeae* pure cultures was carried out using express kits for DNA isolation (Lytekh, Moscow, Russia). DNA was stored at –20°C.

### *N*. *gonorrhoeae* antimicrobial susceptibility testing

Benzylpenicillin and ceftriaxone susceptibility testing of *N*. *gonorrhoeae* isolates and determination of MIC were carried out using the agar dilution method on GC-agar enriched with 1% IsoVitaleX. The obtained MIC values were compared with breakpoints from The European Committee on Antimicrobial Susceptibility Testing (EUCAST) [34].

Isolates tested for susceptibility to benzylpenicillin were categorized as S (susceptible, MIC_pen_ ≤ 0.06 mg/L), I (intermediate, 0.12 < MIC_pen_ ≤ 1 mg/L), and R (resistant, MIC_pen_ > 1 mg/L).

For ceftriaxone, according to the EUCAST criteria, isolates with MIC_cef_ ≤ 0.125 mg/L were considered susceptible, and isolates with MIC_cef_ > 0.125 mg/L were considered resistant.

For comparison, the US Clinical and Laboratory Standards Institute (CLSI) criteria [35] were also used. The CLSI criteria are less strict: *N*. *gonorrhoeae* isolates are considered penicillin resistant if MIC_pen_ ≥ 2 mg/L; for ceftriaxone, susceptible strains are strains with MIC_cef_ ≤ 0.25 mg/L.

All *N*. *gonorrhoeae* isolates were tested for the presence of beta-lactamases by a nitrocefin test using nitrocefin discs (Cefinase, bioMérieux).

### Genetic analysis of *N*. *gonorrhoeae*

#### Identification of genetic determinants of antimicrobial resistance

The detection of genetic determinants of *N*. *gonorrhoeae* resistance to antimicrobials was carried out using a hydrogel low-density oligonucleotide microarray. The microarray was previously developed for the identification of causative agents of human reproductive tract infections, including *N*. *gonorrhoeae*, and for the simultaneous detection of genetic markers of resistance to different antimicrobial drugs [36]. The microarray consisted of elements with immobilized oligonucleotides for the detection of different mutations and other determinants associated with resistance to beta-lactams: mutations in *penA* resulting in the insertion of Asp in the 345 position in PBP2 (insAsp345), mutations in *ponA* resulting in the amino acid substitution Leu421Pro in PBP1, the *bla*_TEM_ plasmid and Met182Thr and Gly238Ser substitutions in the gene encoding beta-lactamase, mutations in *porB* leading to the amino acid changes Gly120Lys/Asp/Asn/Thr and Ala121/Asp/Asn/Gly/Ser in the porin protein, and deletion A (delA) and insertions T and TT (insT and insTT) in the promoter region of *mtrR*.

The microarray also allowed the simultaneous identification of mutations associated with resistance to other antimicrobials [1,12,36]: fluoroquinolones (mutations in *gyrA* and *parC*), tetracyclines (mutations in the 16S rRNA and *rpsJ*, plasmid *tetM*), macrolides (mutations in the 23S rRNA and *mefA*), and spectinomycin (mutations in the 16S rRNA). All the results obtained with the microarray are presented in S1 Table, although only the genetic determinants of resistance to beta-lactam antibiotics are discussed in this paper.

For isolates with reduced susceptibility to ceftriaxone, the sequence of *penA* was determined using a 3730xl Genetic Analyzer (Applied Biosystems, USA).

Sequencing of *porB* and *tbp* for *N*. *gonorrhoeae* multiantigen sequence typing (NG-MAST) was performed according to a conventional protocol [37] using a 3730xl Genetic Analyzer.

#### Determination of *bla*_TEM_ plasmid types and beta-lactamase variants

The type of *bla*_TEM_ plasmid was determined by multiplex PCR followed by electrophoresis in a 1% agarose gel. PCR was carried out with the primers previously described by Palmer et al. [38]: BL1, 5′-TACTCAATCGGTAATTGGCT-3′; BL2, 5′-CACCTATAAATCTCGCAAG C-3′; BL3, 5′-CCATAGTGTTGAGTATTGCGAA-3′; BL4, 5′-TCATTCGTGCGTTCTAGGA-3′. The PCR product sizes were BL2 + BL3 = 958 bp (Asian plasmid), BL1 + BL3 = 1191 bp (African plasmid), and BL2 + BL4 = 650 bp (Toronto/Rio plasmid).

The presence of mutations in the beta-lactamase gene of *N*. *gonorrhoeae* that result in Met182Thr, Ala185Thr, and Gly238Ser substitutions was checked by sequencing with the primers ^6617^GGCACTGGTGCAACGGAAAT^6636^ and ^446^GGTCTGACGCTCAGTGGAAC^465^, GenBank ID NC_002098.1.

### Statistical analysis

The significance of the differences between groups was assessed using a non-parametric Kruskal-Wallis test (significance level α < 0.05) in IBM SPSS Statistics V23 software. Then, multiple pairwise comparisons of groups (with the control group) were carried out using Dunn’s Q criterion. Dunn’s criteria were calculated, and p values were determined using previously defined critical values [39]. The critical value for the Q criterion was 3.90 for the number of groups under study.

#### Phylogenetic analysis of nucleotide sequences

The Bayesian information criterion was used for the selection of the nucleotide substitution models in MEGA7 software [40]. For the NG-MAST gene locus, the evolutionary history was inferred using the maximum likelihood method based on the Hasegawa-Kishino-Yano model [41] with invariant sites. The initial tree(s) for the heuristic search were obtained automatically by applying the neighbour-joining and BIONJ algorithms to a matrix of pairwise distances estimated using the maximum composite likelihood approach and then selecting the topology with the superior log likelihood value. The tree was drawn to scale with branch lengths measured in number of substitutions per site.

## Results

### Penicillin resistance in *N*. *gonorrhoeae* isolates

The analysis of phenotypic characteristics in the recent (2015–2017) population of gonococcal infections in Russia revealed 40 isolates (7.7%) resistant to benzylpenicillin (with MIC_pen_ > 1 mg/L), 248 isolates (47.5%) with intermediate susceptibility (MIC_pen_ 0.12–1 mg/L) and 234 (44.8%) susceptible isolates. Microarray hybridization and sequencing revealed 396 isolates that bore different determinants associated with resistance to penicillins, including 25 isolates carrying the *bla*_TEM_ plasmid (Table 1). The isolate characteristics of the obtained dataset (522 samples), including susceptibility (MIC values), mutations revealed using microarrays and results of NG-MAST typing, are summarized in S1 Table (in addition to penicillins, the microarray results include detection of resistance determinants to fluoroquinolones, tetracyclines, azithromycin, and spectinomycin). Isolates carrying chromosomal mutations demonstrated mostly intermediate susceptibility to benzylpenicillin, whereas isolates with the *bla*_TEM_ plasmid had high levels of resistance (Table 1), confirming the previously described correlation between the presence of plasmid beta-lactamases and a high level of penicillin resistance in *N*. *gonorrhoeae* [26,42].

**Table 1:** Genetic determinants and susceptibility of *N*. *gonorrhoeae* isolates to benzylpenicillin. Mutations: *penA*–ins345Asp, *ponA–*Leu421Pro, *mtrR* (promoter region)–-35delA, *porB*–Gly120Lys/Asp/Asn/Thr and/or Ala121/Asp/Asn/Gly/Ser.

№	Mutations in genes	MIC_pen_ (mg/L) / number of isolates with the corresponding MIC_pen_											Median MIC_pen_ (mg/L)	Number of susceptible (S), intermediate (I) and resistant (R) isolates				Comparison with the wild-type isolate^c^	
		0.015	0.03	0.06	0.12	0.25	0.5	1	2	8	16	≥ 32		S (≤ 0.06)	I (0.12–1)	R (> 1)	Total	Dunn’s criterion Q	*p* value
1	No mutations^a^	85	13	11	11	4	2	–	–	–	–	–	0.015	109	17	–	126	–	–
2	*penA*	20	17	40	34	14	4	1	1	–	–	–	0.06	77	53	1	131	5.45	< 0.001
3	*ponA*	–	–	1	–	2	–	–	–	–	–	–	0.25	1	2	–	3	2.08	> 0.5
4	*mtrR*	–	1	–	–	1	–	–	–	–	–	–	0.14	1	1	–	2	1.21	> 0.5
5	*porB*	2	–	1	–	–	2	–	–	–	–	–	0.06	3	2	–	5	1.69	> 0.5
6	*penA* and *mtrR*	2	2	1	1	1	–	–	1	–	–	–	0.045	5	2	1	8	1.83	> 0.5
7	*ponA* and *mtrR*	1	–	–	–	1	–	1	–	–	–	–	0.25	1	2	–	3	2.01	> 0.5
8	*penA* and *ponA*	3	4	11	13	14	6	1	1	–	–	–	0.12	18	34	1	53	6.69	< 0.001
9	*penA* and *porB*	1	–	3	3	6	6	2	–	–	–	–	0.25	4	17	–	21	6.15	< 0.001
10	*penA*, *ponA*, and *mtrR*	2	4	–	3	7	13	9	–	–	–	–	0.5	6	32	–	38	8.69	< 0.001
11	*penA*, *ponA*, and *porB*	–	1	4	2	14	7	9	3	–	–	–	0.25	5	32	3	40	9.48	< 0.001
12	*ponA*, *mtrR*, and *porB*	–	–	–	–	–	–	2	1	–	–	–	1	–	2	1	3	4.12	< 0.001
13	*penA*, mtrR, and *porB*	2	–	–	1	3	3	1	1	–	–	–	0.25	2	8	1	11	4.58	< 0.001
14	*penA*, *ponA*, *mtrR*, and *porB*	–	–	2	4	5	22	13	7	–	–	–	0.5	2	44	7	53	12.13	< 0.001
15	Presence of *bla*_TEM_^b^									1	8	16	≥ 32	–	–	25	25	12.17	< 0.001
	Total number of isolates													234	248	40	522		

^a^ No mutations in chromosomal genes and no *bla*_TEM_ plasmids were found.

^b^ Mutations in chromosomal genes are not indicated here for isolates with *bla*_TEM_.

Mutations in *penA* and *ponA* were predominant among the determinants that affected resistance. The most frequent mutation in the samples was the insertion of aspartic acid at codon 345 of *penA*, both as a single mutation and in combination with other changes. The ins345Asp mutation was observed in 378 of 522 isolates (72.4%). A single ins345Asp mutation did not result in the appearance of benzylpenicillin-resistant isolates, but a statistically significant increase in the median MIC_pen_ to 0.06 mg/L was observed. The Leu421Pro substitution in *ponA* was found in 204 isolates (39.0%). This mutation had a more pronounced effect on benzylpenicillin resistance, resulting in the formation of intermediately susceptible isolates with a median MIC_pen_ of 0.25 mg/L (Table 1).

The deletion of adenine (delA) in the promoter region of *mtrR* was identified in 119 *N*. *gonorrhoeae* isolates (22.8%), whereas the insertions described in the literature of thymidine (T) or TT at the -10 position of *mtrR* were not found.

Mutations in *porB* were revealed in 140 isolates (26.8%). Substitutions in PorB in the presence of simultaneous mutations in *mtrR* led to an increase in the median MIC_pen_ to 0.25–0.5 mg/L (isolates with intermediate susceptibility). However, there was no statistically relevant difference in resistance level depending on the type of amino acid change at residues 120 and 121 (data not shown).

As a rule, compared with single mutations, the accumulation of several mutations resulted in an increase in the resistance of *N*. *gonorrhoeae* isolates: statistically significant differences in median MIC_pen_ values were obtained (Table 1). Hence, the median MIC_pen_ for the isolates with mutations in two genes increased to 0.25 mg/L, and the simultaneous presence of mutations in four genes (53 samples) led to an increase in the median MIC_pen_ to 0.5 mg/L. Table 1 shows Dunn’s Q criterion for the comparison of groups of isolates carrying mutations with the group of wild-type isolates (groups 2–15 compared to group 1). Some scores were found to be statistically non-significant due to the small numbers of samples in groups.

To determine whether the differences in the median MIC_pen_ for the sample groups with chromosomal mutations (groups 2–14 in Table 1) were statistically significant, additional pairwise comparisons of groups were carried out. High values of the Q criterion were obtained for group 2 (single mutation in *penA*) compared with group 10 (mutations in *penA*, *ponA*, and *mtrR*), group 2 compared with group 11 (mutations in *penA*, *ponA*, and *porB*), group 2 compared with group 14 (mutations in *penA*, *ponA*, *mtrR*, and *porB*), and group 8 (mutations in *penA* and *ponA*) compared with group 14 (mutations in *penA*, *ponA*, *mtrR*, and *porB*). These results indicate that the accumulation of mutations, *i*.*e*., mutations in 3 or 4 genes compared with mutations in 1 or 2 genes, led to statistically significant increases in MIC_pen_.

The presence of the *bla*_TEM_ plasmid was detected in 25 (4.8%) *N*. *gonorrhoeae* isolates. All isolates with the *bla*_TEM_ plasmid demonstrated resistance to benzylpenicillin with MIC_pen_ > 8 mg/L; 24 isolates had a MIC_pen_ ≥ 16 mg/L (Tables 1 and 2), regardless of mutations in chromosomal genes.

**Table 2:** Characterization of *N*. *gonorrhoeae* isolates carrying *bla*_TEM_ plasmids.

Region	NG-MAST type	MIC_pen_, mg/L	Type of *bla*_TEM_ plasmid	Beta-lactamase variant	Presence of *tetM* plasmid	MIC_tet_, mg/L
Arkhangelsk (2 isolates)	14604	16	African	TEM-1	*tetM*	32
Arkhangelsk	12096	16	African	TEM-1	*tetM*	32
Astrakhan	14596	16	African	TEM-1	*tetM*	32
Bryansk (7 isolates)	14826	≥ 32	African	TEM-1	*tetM*	16
Chuvashiya	10158	≥ 32	Toronto/Rio	TEM-135	*tetM*	8
Kaluga (3 isolates)	15644	≥ 32	African	TEM-1	*tetM*	64
Kaluga	15644	≥ 32	African	TEM-1	*–*	1
Kaluga	16173	≥ 32	Toronto/Rio	TEM-135	*–*	1
Kaluga	12096	16	African	TEM-1	*tetM*	32
Moscow	3109	≥ 32	African	TEM-1	*–*	0.5
Moscow	7848	16	African	TEM-1	*tetM*	32
Moscow	3109	8	African	TEM-1	*–*	0.25
Novosibirsk	15748	≥ 32	African	TEM-1	*tetM*	64
Omsk	14826	≥ 32	African	TEM-1	*tetM*	8
Ryazan	13336	16	African	TEM-1	*tetM*	32
Ryazan	14015	16	African	TEM-1	*tetM*	32

The type of *bla*_TEM_ plasmid and the variant of beta-lactamase were identified for the first time in the samples collected in the Russian Federation. The majority (23 of 25) of *bla*_TEM_ plasmids were of the African type, the most widespread type in the world. Two plasmids were of the Toronto/Rio type. Interestingly, the penicillinase-producing strains in neighbouring Poland contained both the African and Toronto/Rio plasmids (50/50) [26].

The African-type plasmids contained a TEM-1 beta-lactamase gene with a Met residue at position 182, and both Toronto/Rio plasmids carried a TEM-135 beta-lactamase gene with a Met182Thr substitution that was in accordance with previously described data [24,26]. Mutations that can result in the emergence of beta-lactamase activity towards cephalosporins were not found in the analysed *N*. *gonorrhoeae* isolates.

**Fig 1:**
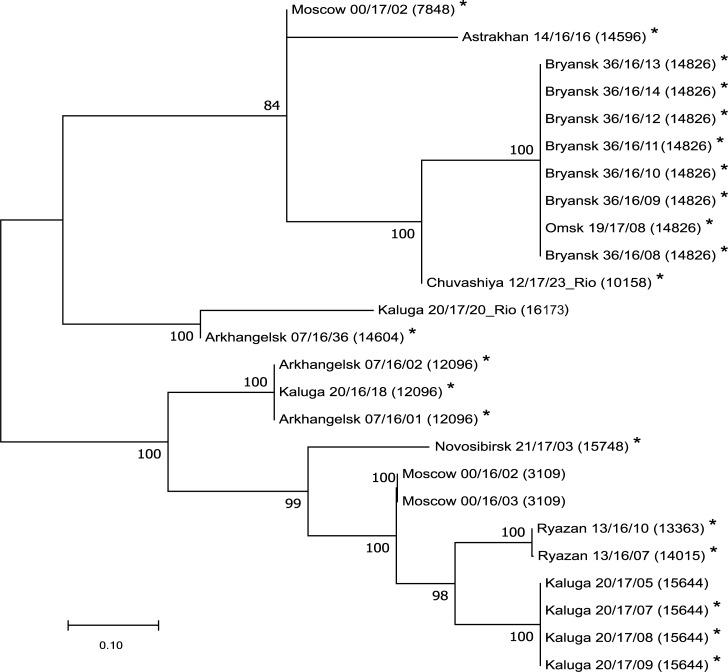
Phylogenetic tree constructed with the NG-MAST gene loci of *N*. *gonorrhoeae* isolates collected in the Russian Federation in 2015–2017 and carrying *bla*_TEM_ plasmids. Bootstrap values are shown next to the branches. The origin of each isolate and its sample code are indicated. Isolates harbouring *bla*_TEM_ (resistance to penicillins) and *tetM* (resistance to tetracycline) plasmids simultaneously are marked with asterisks.

For the *N*. *gonorrhoeae* isolates carrying *bla*_TEM_ plasmids, a maximum likelihood phylogenetic tree was constructed for the loci used for NG-MAST typing (Fig 1). According to the phylogenetic results, the isolates with *bla*_TEM_ plasmids can be divided into three clusters. The isolates with Toronto/Rio plasmids were located in different clusters. Isolates from nearby regions were often closer to each other than isolates from distant regions, with some exceptions (Arkhangelsk, Kaluga, and Moscow). These results indicate that several parallel processes can be observed: horizontal gene transfer, vertical gene transfer, and migration of people with *N*. *gonorrhoeae*.

### Simultaneous presence of plasmids associated with resistance to benzylpenicillin and tetracycline

Twenty-two of the 25 *N*. *gonorrhоeae* isolates harboured the *bla*_TEM_ plasmid, and the plasmid with *tetM* was responsible for high resistance to tetracyclines (MIC_tet_ > 8 mg/L) [43] (Table 2). The *tetM* in *N*. *gonorrhoeae* is located on a large (~25 MDa) conjugative plasmid and, as previously shown [44–47], the backbone of this plasmid mobilizes the transfer of the small gonococcal beta-lactamase plasmids (3–6 MDa depending on the plasmid type) to other *N*. *gonorrhoeae* strains and other *Neisseria* species, *i*.*e*., it may facilitate the transfer of other plasmids carrying other drug resistance markers into the cell. Because tetracycline was previously actively used for the treatment of gonorrhoea throughout the world, the level of resistance to this drug remains very high. In Russia, 29% of *N*. *gonorrhoeae* isolates were tetracycline resistant in 2015–2017, and one-quarter of these isolates contained the plasmid with *tetM* [43]. As the presence of the *tetM* plasmid facilitates the acquisition of other plasmids by the cell, there is a danger of the appearance of multiresistant *N*. *gonorrhoeae* species with high plasmid-mediated resistance.

### Ceftriaxone resistance in *N*. *gonorrhoeae* isolates

Only three isolates with decreased susceptibility to ceftriaxone were found in the studied samples collected in Russia in 2015–2017. One isolate showed MIC_cef_ = 0.25 mg/L; this isolate is considered resistant according to the EUCAST criteria but susceptible according to the CLSI criteria. Another two isolates had MIC_cef_ = 0.125 mg/L, and the other 519 isolates had MIC_cef_ values in the range of 0.001–0.06 mg/L. Detailed characteristics of the *N*. *gonorrhoeae* isolates with MIC_cef_ ≥ 0.125 mg/L are presented in Table 3. The distributions of the ceftriaxone MICs and detected genetic determinants in *N*. *gonorrhoeae* isolates are shown in S2 Table.

**Table 3:** Characterization of the *N*. *gonorrhoeae* isolates with the highest ceftriaxone MICs.

№*	Region (year)	Sample code	NG-MAST type	MICcef, mg/L	MICpen, mg/L	Chromosomal genetic determinants				blaTEM plasmid	Type of PBP2 encoded by penA
						penA	ponA	porB	mtrR		
1 (135)	Arkhangelsk (2015)	07/15/49	9480	0.25 (R)	0.25 (I)	insAsp345	no mutations	Gly120Asp	no mutations	–	I
2 (78)	Arkhangelsk (2016)	07/16/42	9486	0.125	1 (I)	insAsp345	no mutations	no mutations	no mutations	–	I
3 (306)	Kaluga (2017)	20/17/05	15644	0.125	≥ 32 (R)	insAsp345	Leu421Pro	no mutations	no mutations	*bla*_TEM-1_	XVI

* The isolate number in S1 Table is indicated in brackets.

All these isolates carried the Asp insertion in the 345 position of *penA*. Additional sequencing of *penA* revealed a non-mosaic structure for all three isolates. The protein sequences encoded by *penA* in these samples were homologous and belonged to types I and XVI; the amino acid changes that are present in cephalosporin-resistant isolates were not typical of these structure types [16,17]. The chromosomal mutations identified in these isolates were associated with resistance or intermediate resistance to benzylpenicillin; however, they cannot explain the mechanism of ceftriaxone MIC elevation for these isolates.

**Fig 2:**
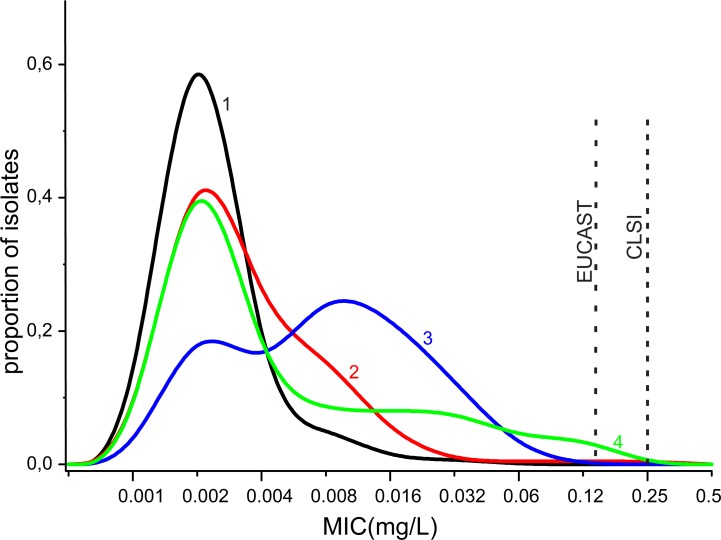
Ceftriaxone MIC distributions of *N*. *gonorrhoeae* isolates with different genetic determinant profiles. Wild-type isolates (1), isolates with mutations in one or two chromosomal genes (2), isolates with mutations in three or four chromosomal genes (3), and isolates carrying *bla*_TEM_ plasmids (4) are shown.

The distribution of mutations in the whole pool of isolates (Fig 2, S2 Table) indicated that mutations in a single gene or simultaneous mutations in two genes (*penA*, *ponA*, *mtrR* (promoter region), and *porB*) did not result in a change in the median MIC_cef_ in comparison with the wild-type isolates (MIC_cef_ = 0.002–0.003 mg/L). A statistically significant increase in MIC_cef_ to 0.004–0.008 mg/L was observed in the presence of three simultaneous mutations in *penA*, *ponA*, and *mtrR* or *penA*, *ponA*, and *porB*. The occurrence of mutations in four chromosomal genes led to an increase in MIC_cef_ to 0.015 mg/L (Fig 2). The accumulation of mutations also resulted in an increase in MIC_cef,_ but this increase did not reach the MIC level of ceftriaxone-resistant isolates.

Thus, a number of isolates with decreased susceptibility to ceftriaxone were found among recent *N*. *gonorrhoeae* isolates in Russia, and the analysis did not reveal mutations associated with resistance to third-generation cephalosporins.

## Discussion

In this work, the phenotypic susceptibility and genetic determinants of resistance to benzylpenicillin and ceftriaxone were analysed in *N*. *gonorrhoeae* clinical isolates collected in Russia in 2015–2017. The low-density oligonucleotide microarray [36] used in this work proved to be a useful and convenient tool for the rapid screening of drug resistance determinants. The limitation of this assay was the restricted number of identified genetic markers relevant to antibiotic resistance. Hence, the microarray did not allow detection of mosaic *penA* alleles and mutations in mosaic alleles due to the large number of alterations (more than 70 mutations are known to date) and the presence of several SNPs in non-mosaic *penA* alleles. Therefore, *penA* was additionally analysed by sequencing.

The recent results of *N*. *gonorrhoeae* surveillance in Russia within the framework of the RU-GASP Programme [48] indicated decreasing trends in resistance to the antibiotics previously used for gonorrhoea treatment (benzylpenicillin, tetracycline, and ciprofloxacin). However, the level of resistance to these antibiotics remains high, excluding the possibility of reviving their therapeutic use for gonococcal infection. Isolates with slightly decreased susceptibility to ceftriaxone appeared only sporadically [48]. Among the clinical isolates collected in 2015–2017 in Russia and analysed in this work, 7.7% were resistant to benzylpenicillin, and 47.5% showed intermediate resistance. The accumulation of mutations in chromosomal genes (*penA*, *pon*, *porA*, and *mtrR*) led to a stepwise increase in penicillin MIC to values characteristic of intermediate resistance (up to 0.5 mg/L).

An additional limitation for penicillin usage is the presence of a *bla*_TEM_ plasmid that is potentially capable of rapid horizontal transfer in the case of selective pressure related to this antibiotic. Notably, the ratio of plasmid penicillinase-producing *N*. *gonorrhoeae* isolates in Russia was 4.8%, which is lower than the average ratio of 14.9% reported for Euro-GASP countries [49].

The study of susceptibility to another beta-lactam antibiotic, ceftriaxone, showed a high indication of susceptibility in the Russian isolates collected in 2015–2017, which is a good reason to maintain the recommendation to use ceftriaxone as a first-line drug for gonorrhoea therapy. It should be noted that the Euro-GASP report indicated stable overall resistance levels to third-generation cephalosporins, both cefixime and ceftriaxone, in European countries at the present time [49]. Only one isolate with MIC_cef_ = 0.25 mg/L, which is considered resistant according to the EUCAST criteria, was found among the samples collected in Russia in 2015–2017. Two isolates had MIC_cef_ at the resistance breakpoint (0.125 mg/L). The analysis of chromosomal determinants indicated their roles in the shift of MIC_cef_ towards increased values, especially with the simultaneous presence of mutations in the target genes (*penA* and *ponA*) and the drug delivery (*porB*) and efflux (*mtrR*) systems. Additional analysis of samples with maximum MIC_cef_ values, including sequencing of *penA*, did not reveal the mutations associated with resistance to third-generation cephalosporins and showed a non-mosaic structure of *penA*. It is worth noting that five *N*. *gonorrhoeae* samples with a non-mosaic *penA* allele and decreased susceptibility to extended-spectrum cephalosporins (MIC_cef_ = 0.5 mg/L) were found among isolates collected in the USA; it was proposed that the observed phenotype might have resulted from the combined effects of mutations in multiple genes [50].

One of the interesting facts observed in this work was the simultaneous presence of the *bla*_TEM_ and *tetM* plasmids associated with high resistance to penicillins and tetracyclines in *N*. *gonorrhoeae* isolates. Previous studies [45–47] have shown that the conjugative *tetM* plasmid in *N*. *gonorrhoeae* facilitates the acquisition of other plasmids by the cell. This manner of developing drug resistance should not be underestimated. Thus, analysis of drug resistance determinants in *N*. *gonorrhoeae* calls for special attention to isolates resistant to tetracyclines and carrying *tetM* plasmids, because the presence of this genetic element simplifies the transfer of *bla*_TEM_ plasmids with penicillin resistance markers and other plasmids containing genes associated with resistance to other antimicrobial drugs.

## Supporting information

S1 Table Characteristics of *N*. *gonorrhoeae* clinical isolates used in this study, including the results of drug susceptibility testing, profiles of genetic determinants of drug resistance and NG-MAST sequence types. ST–NG-MAST sequence type, Pen–penicillin, Tet–tetracycline, Cef–ceftriaxone, Cip–ciprofloxacin, Spec–spectinomycin, Azit–azithromycin.
(XLSX)

S2 Table Genetic determinants and susceptibility of *N*. *gonorrhoeae* isolates to ceftriaxone. Mutations: *penA*–ins345Asp, *ponA–*Leu421Pro, *mtrR* (promoter region)–-35delA, *porB*–Gly120Lys/Asp/Asn/Thr and/or Ala121/Asp/Asn/Gly/Ser.
(DOCX)
