# Supplementary material for: Resistance of Neisseria gonorrhoeae isolates to beta-lactam antibiotics (benzylpenicillin and ceftriaxone) in Russia, 2015–2017
Source: PLoS One. 2019 Jul 25;14(7):e0220339. doi: 10.1371/journal.pone.0220339 (PMC6657886; doi:10.1371/journal.pone.0220339)
Supplement: S2 Table — Mutations: penA–ins345Asp, ponA–Leu421Pro, mtrR (promoter region)–-35delA, porB–Gly120Lys/Asp/Asn/Thr and/or Ala121/Asp/Asn/Gly/Ser. (DOCX) [file pone.0220339.s002.docx]

**S2 Table. Genetic determinants and susceptibility of *N. gonorrhoeae* isolates to ceftriaxone**

Mutations: *penA* – ins345Asp, *ponA* – Leu421Pro, *mtrR* (promoter region) – -35delA, *porB* – Gly120Lys/Asp/Asn/Thr and/or Ala121/Asp/Asn/Gly/Ser

| Mutations in genes | MIC_cef_ (mg/L) / number of isolates with the corresponding MIC_cef_ | | | | | | | | | Median  (mg/L) | Comparison with the wild-type | |
| --- | --- | --- | --- | --- | --- | --- | --- | --- | --- | --- | --- | --- |
|  | 0.001 | 0.002 | 0.004 | 0.008 | 0.016 | 0.032 | 0.06 | **0.125** | **0.250** |  | Dunn criterion Q | *p* value |
| No mutations^a^ | 0 | 110 | 9 | 7 | 1 | 1 | 0 | 0 | 0 | 0.002 |  |  |
| *penA* | 1 | 83 | 26 | 26 | 5 | 0 | 0 | **1** | 0 | 0.002 | 3.67 | < 0.01 |
| *ponA* | 0 | 2 | 1 | 0 | 0 | 0 | 1 | 0 | 0 | 0.003 | 1.52 | > 0.5 |
| *mtrR* | 0 | 1 | 1 | 0 | 0 | 0 | 0 | 0 | 0 | 0.003 | 0.59 | > 0.5 |
| *porB* | 0 | 4 | 0 | 1 | 0 | 0 | 0 | 0 | 0 | 0.002 | 0.32 | > 0.5 |
| *penA* and *mtrR* | 0 | 7 | 1 | 0 | 0 | 0 | 0 | 0 | 0 | 0.002 | 0.19 | > 0.5 |
| *ponA* and *mtrR* | 0 | 1 | 2 | 0 | 0 | 0 | 0 | 0 | 0 | 0.004 | 1.09 | > 0.5 |
| *penA* and *ponA* | 0 | 30 | 15 | 7 | 3 | 0 | 1 | **1** | 0 | 0.002 | 3.53 | < 0.01 |
| *penA* and *porB* | 0 | 9 | 6 | 3 | 2 | 1 | 0 | 0 | **1** | 0.004 | 3.64 | < 0.005 |
| *penA*, and *ponA*, and *mtrR* | 0 | 12 | 4 | 13 | 6 | 2 | 1 | 0 | 0 | 0.008 | 6.13 | < 0.001 |
| *penA*, and *ponA*, and *porB* | 0 | 17 | 8 | 11 | 8 | 1 | 0 | 0 | 0 | 0.004 | 5.42 | < 0.001 |
| *ponA*, and *mtrR*, and *porB* | 0 | 0 | 0 | 1 | 1 | 1 | 0 | 0 | 0 | 0.015 | 3.51 | < 0.01 |
| *penA*, and *mtrR*, and *porB* | 0 | 3 | 0 | 5 | 1 | 2 | 0 | 0 | 0 | 0.008 | 4.19 | < 0.001 |
| *penA*, and *ponA*, and *mtrR*, and *porB* | 0 | 3 | 6 | 11 | 18 | 14 | 2 | 0 | 0 | 0.015 | 11.46 | < 0.001 |
| Presence of *bla*_TEM_^b^ | 0 | 14 | 3 | 2 | 2 | 2 | 1 | 1 | 0 | 0.002 | 3.16 | < 0.01 |

^a^ No mutations in chromosomal genes and no *bla*_TEM_ plasmids were found.

^b^ Mutations in chromosomal genes are not indicated here for isolates with *bla*_TEM_.
